# Quantification of Shared Air: A Social and Environmental Determinant of Airborne Disease Transmission

**DOI:** 10.1371/journal.pone.0106622

**Published:** 2014-09-02

**Authors:** Robin Wood, Carl Morrow, Samuel Ginsberg, Elizabeth Piccoli, Darryl Kalil, Angelina Sassi, Rochelle P. Walensky, Jason R. Andrews

**Affiliations:** 1 Desmond Tutu HIV Centre, Institute of Infectious Diseases and Molecular Medicine, and Department of Medicine, University of Cape Town Faculty of Health Sciences, Cape Town, South Africa; 2 Department of Electrical Engineering, Faculty of Engineering & the Built Environment, University of Cape Town, Cape Town, South Africa; 3 Center for AIDS Research, Harvard Medical School, Boston, Massachusetts, United States of America; 4 Division of Infectious Diseases and Geographic Medicine, Stanford University School of Medicine, Stanford, California, United States of America; Fondazione Bruno Kessler, Italy

## Abstract

**Background:**

Tuberculosis is endemic in Cape Town, South Africa where a majority of the population become tuberculosis infected before adulthood. While social contact patterns impacting tuberculosis and other respiratory disease spread have been studied, the environmental determinants driving airborne transmission have not been quantified.

**Methods:**

Indoor carbon dioxide levels above outdoor levels reflect the balance of exhaled breath by room occupants and ventilation. We developed a portable monitor to continuously sample carbon dioxide levels, which were combined with social contact diary records to estimate daily rebreathed litres. A pilot study established the practicality of monitor use up to 48-hours. We then estimated the daily volumes of air rebreathed by adolescents living in a crowded township.

**Results:**

One hundred eight daily records were obtained from 63 adolescents aged between 12- and 20-years. Forty-five lived in wooden shacks and 18 in brick-built homes with a median household of 4 members (range 2–9). Mean daily volume of rebreathed air was 120.6 (standard error: 8.0) litres/day, with location contributions from household (48%), school (44%), visited households (4%), transport (0.5%) and other locations (3.4%). Independent predictors of daily rebreathed volumes included household type (p = 0.002), number of household occupants (p = 0.021), number of sleeping space occupants (p = 0.022) and winter season (p<0.001).

**Conclusions:**

We demonstrated the practical measurement of carbon dioxide levels to which individuals are exposed in a sequence of non-steady state indoor environments. A novel metric of rebreathed air volume reflects social and environmental factors associated with airborne infection and can identify locations with high transmission potential.

## Introduction

South Africa has one of the highest population notification rates of tuberculosis (TB) in the world with approximately 1% of population diagnosed with TB disease each year [1], [2]. The annual risk of infection of children in Cape townships has remained high for decades, [3] and currently 5% to 8% of township adolescents become TB-infected each year [4]–[6]. A majority of the Cape Town population therefore becomes TB-infected before adulthood [4]–[6]. Molecular epidemiologic evidence indicates that most infections occur outside of households [7], [8]; however, the specific locations where TB transmission is occurring remain undefined.

The contribution of social deprivation to endemic TB has been debated both before and after *Mycobacterium tuberculosis* was identified as the etiologic agent causing TB [9]–[11]. In the 1950's, the work of Riley and Wells defined TB transmission on a purely physical basis related to the volume of air respired by a susceptible individual and the concentration of exhaled quanta capable of establishing infection [12], [13]. Infectious quanta are micronuclei (<4 microns), which remain airborne and survive for prolonged periods, diffuse throughout indoor spaces and are diluted by infection-free ventilation [12], [13]. In poor communities with high TB prevalence, social interactions frequently occur in crowded and poorly ventilated indoor locations resulting in high probability of TB transmission [14].

Several studies have used the Riley and Wells model to estimate TB transmission risks in specific single locations (e.g. hospital wards) [15]–[20]. However, estimation of contributions from multiple locations to TB infection risk is complex as the exposure time, social-mixing and ventilation differ in each location. Cape township social contacts occur in a variety of indoor locations including households, school classrooms, work places and public

Transportation [21], [22]. Therefore, TB transmission risk may be determined by the quantity of infected air respired in each location.

Carbon dioxide (CO_2_) is a natural tracer gas produced during normal human respiration. Exhaled breath contains approximately 40 000 parts per million (ppm) of CO_2_ compared with approximately 400 ppm in outdoor air [23]. Our study location in Masiphumelele, a township located 40 km from Cape Town, had an average level of 390.8 ppm of CO_2_ in 2012 (IQR: 389.5–391.47) [24]. In the absence of other sources, indoor CO_2_ levels reflect exhaled breath (respiration) and air exchange (ventilation) [25], [26]. Rudnick and Milton demonstrated that measuring “excess” CO_2_ in indoor air can be used to estimate the fraction of air in each inhalation that has been exhaled from other room occupants, and that the “rebreathed fraction” can estimate risk of infection with airborne particles [25]. The equation derived by Rudnick and Milton expanded upon the work of Wells and Riley and used rebreathed fraction to substitute for the more difficult analysis of room ventilation and size. We postulated that the sum of rebreathed air volumes (RAV) from others during normal indoor activities would allow quantification of the social and environmental factors impacting TB transmission. We therefore developed a portable CO_2_ logging device to continuously measure the levels of CO_2_ to which township adolescents were exposed and to thereby determine RAV in all visited indoor locations during a 24-hour period.

## Materials and Methods

### CO_2_ and Global Positioning System logger

**Figure 1:**
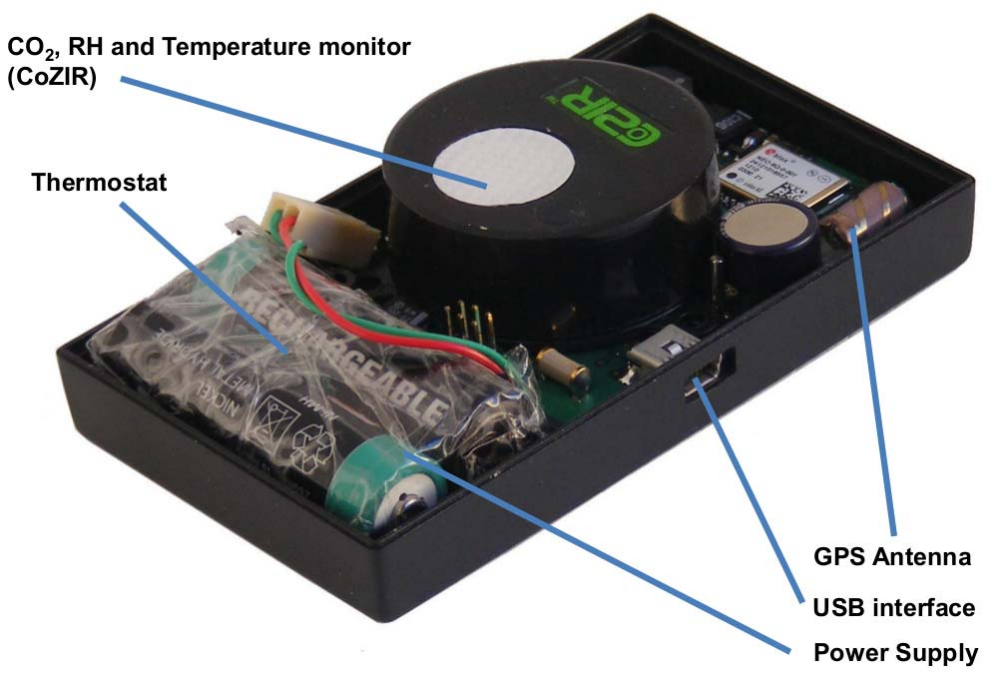
Portable logger to measure CO_2_ concentration, temperature and humidity. An internal view of the portable personal CO_2_ logger incorporated a COZIR Ambient 0–1% transducer (Gas Sensing Solutions Ltd., Glasgow, United Kingdom), GPS sensor, independent power supply and USB interface. Unit dimensions were length 10 cm, width 6 cm and depth 2.5 cm.

A portable logger [Figure 1] was designed to measure CO_2_ concentration, temperature and humidity every 60 seconds, using the COZIR *Ambient 0–1%* transducer (Gas Sensing Solutions Ltd, Glasgow, United Kingdom, http://www.cozir.com/), together with location data captured from a global positioning system (GPS) receiver and time from an onboard, independently powered clock. The logger's dimensions were 10×6×2.5 centimetres and component costs were approximately $250.

The data are delivered to the microcontroller device in serial digital format, which is then stored on flash memory. The microcontroller device can then retrieve flash memory data, on demand, for uploading to a computer via a Universal Serial Bus (USB) port. The logger is powered by Nickel Metal Hydride (NiMH) batteries and includes circuitry for recharging from external power sources. For safety purposes, the electronic circuitry is fused to prevent current in either discharge or charge mode from exceeding safe limits. In addition, a thermostat device has been incorporated to cut the battery from the circuit should battery temperature exceed 70 degrees Celsius. The accuracy of CO_2_ measurements taken by the sensor is ±50 ppm or 3% of each reading (www.cozir.com).

### Time-location diary

A time-location diary was provided to all participants to capture daily routine data including date, location type, time of arrival and departure, and numbers of individuals present for all locations visited. Each diary was filled out continuously, and a field worker clarified incomplete diary entries. The diaries were then entered into a database and were later rechecked by a research assistant. Twenty location types were aggregated to 5 major location categories for data analysis: school/work (daytime activity), transport, own home, other household, and other places. This instrument had been previously used for a social contact study in this community [19].

### Air sampling

Participants underwent training on how to use and recharge the logger, and complete the diary. The logger was attached to a provided lanyard (∼50 cm) or worn in a waist pocket during a 48-hour period. Participants were instructed not to breathe directly into the logger. Sets of more than 1 100 logged environmental data points recorded during any 24-hour period were used in subsequent analyses.

### Pilot study

**Figure 2:**
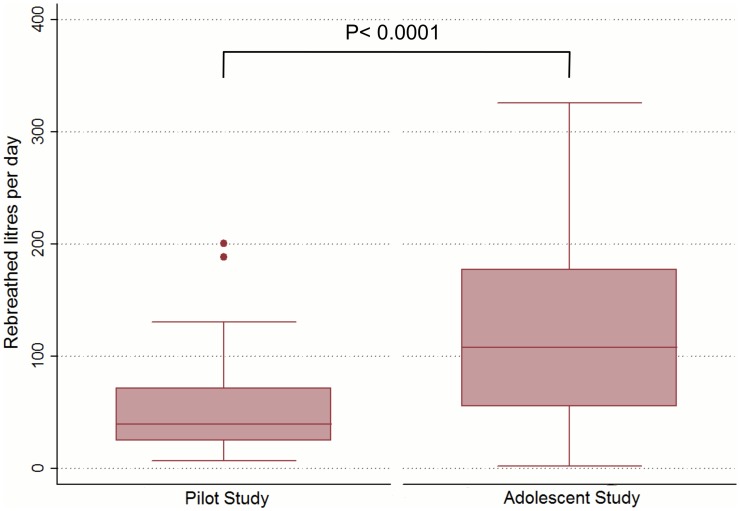
Daily volumes of rebreathed air in the pilot study and the adolescent study. Pilot study (left bar) shows median, inter-quartile ranges and maximum and minimum daily volumes of rebreathed air from others for 17 adults providing 29 daily records. Adolescent study (right bar) shows median, inter-quartile ranges and maximum and minimum daily volumes of rebreathed air of 108 daily records from 63 adolescents living in a high TB prevalence township. N.B. A single outlier value of 550 litres per day for the adolescent study is not shown as it exceeds the maximal value of the ordinate scale.

A pilot study established the practicality of carrying the logger for 48 hours, including position on person, battery recharging procedures and recording of location data in the diary. A heterogeneous sample (15 females and 2 males) with a median age of 39-years (range: 21–63 years) was recruited from the clinical, research and secretarial staff of the Desmond Tutu HIV Centre at the University of Cape Town. The subjects provided 29 daily records with an overall mean RAV of 58.6 (standard error [SE] 11.4) litres per day [Figure 2]. Volume contributions by location were highly variable; mean RAV in transport was 7.1 (SE 3.4) litres per day but with up to 85.5 litres per day recorded in public transport. The location contributions to daily-RAV were 12.4% for transport, 50.3% for own and visited households, 26.8% for workplace and 10.4% for various other locations. The pilot study population was a low TB risk group as no TB diagnoses had been made in the prior 10 years.

### Adolescent study population

A high-TB risk study population of 63 adolescents (37 female, 26 male) with a median age of 17-years (range: 12–20 years) was recruited at the Desmond Tutu Youth Centre in Masiphumelele, Cape Town; a poor community where the annual TB notification rate exceeds 2000 cases per 100 000 [27].

### Data processing and analysis

The data were downloaded as text files and entered into a customised Microsoft Access database. The diary data and times were aligned with the CO_2_ values and corresponding times recorded by the CO_2_ logger. The time period of interest was identified and the rebreathed values were calculated against the lowest CO_2_ value measured in the 24-hour time period. Small gaps in the environmental data capturing were observed and these were filled using an automated algorithm that identified gaps in the trace of more than one minute in length, averaged the starting and ending rebreathed values, multiplied the result by the period of the gap to estimate rebreathed air during the gap. This value was distributed between the beginning and end point of the gap.

Rebreathed proportions were calculated using Rudnick and Milton's equation as shown [25]:



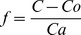
(1)


Where 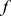 is equivalent to the fraction of air that is exhaled breath, C is the observed concentration of CO_2_ in the indoor air, *Co* is the concentration of CO_2_ in the outdoor air and *Ca* is the concentration of CO_2_ in the exhaled air (estimated from literature) [23]. In other words, the proportion of air that is being rebreathed can be estimated from the excess carbon dioxide observed in the room, divided by the concentration of carbon dioxide in exhaled breath. The outdoor CO_2_ values were defined by the minimum recorded value from each 24-hour record set. For persons at low levels of physical activity, *Ca* was estimated to be 38,000 ppm based on a CO_2_ production rate of 0.31 litres/minute and respiratory minute volume of 8.0 litres/minute [23].

The recorded number of people present in the indoor location was used to estimate the rebreathed proportion from other people:



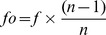
(2)


Here, *n* is the number of people recorded at the indoor location (including the participant). RAV for each 60-second time-period was calculated from the product of *fo* and the minute respiratory volume, *p (8 liters per minute)*, and summed over all observations:
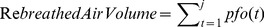
(3)


**Figure 3:**
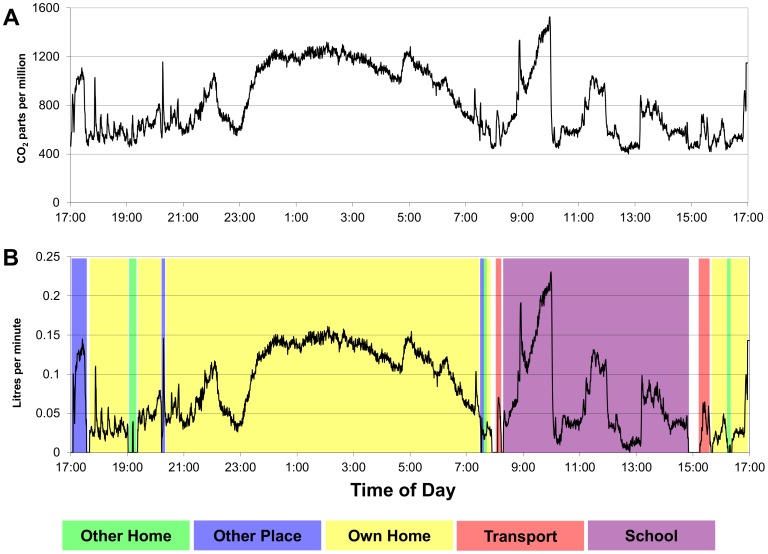
**Figure 3 A**: Ambient parts per million of CO_2_ recorded at minute intervals by the logging device carried by a subject during a 24-hour period. **Figure 3 B**: Litres per minute of rebreathed air with additional allocation to specific locations. Litres per minute of rebreathed air were calculated for a 24-hour period (transformation from ambient CO_2_ levels in Figure 2A) and additionally allocated to specific locations using diary and GPS information. The volume of rebreathed shared air is represented by the area under the curve for each location visited and the daily rebreathed volume is the sum of all volumes at all locations visited.

Thus, continuously recorded ambient CO_2_ values [Figure 3A] can be transformed (using equations 1 and 2) into continuous measures of rebreathed (shared) air at different visited locations [Figure 3B]. The RAV for any time-period was the sum of the 60-second rebreathed volumes accruing in that time period equal to the area under the curve of rebreathed air for the time-period of interest [Figure 3B].

We examined determinants of RAV through linear, mixed-effects, multilevel bivariate and multivariate models, including age, sex, housing type (shack/brick), season, and the number of individuals in household and sleeping space. To account for correlation in multiple, nested observations of the same individual on different days, we used a two-level model with individuals and observations. Season was dichotomized into colder months (May-October) and warmer months (November-April).[28] Because rebreathed litres were non-normally distributed, we log-transformed rebreathed litres, which reduced the skewness and kurtosis and improved the normality of the regression residuals. We further examined residual plots for the predicted, transformed dependent variables. For multivariable analyses, we used Allen-Cady, modified backward selection procedure. In this procedure, we pre-specified forced variables for inclusion (age and sex) and then used a threshold p-value of 0.20 for removal of variables of least importance. Ultimately, all considered variables were found to be under this p-value threshold and were retained [29]. We calculated conditional goodness-of-fit for the mixed-effects model using the approach of Nakagawa and Schielzeth [30]. We also used a multilevel model as above to compare rebreathed litres between adults (pilot study) and students. Statistical analyses were performed using Stata 11.0 (StataCorp, College Station, Texas, USA).

### Ethics Statement

For adults, written informed consent for participation in the study was obtained while for minors, written informed assent was obtained along with written informed consent from a parent or guardian. The Human Research Ethics Committee of the Faculty of Health Sciences at the University of Cape Town approved the study.

## Results

### Township adolescent study

Subjects were all residents of the township, and 45 (71%) lived in a wooden shack and 18 (29%) in brick-built house. The median household size was 4 individuals (range: 4–9) and the median number of individuals sharing sleeping quarters was 2 (range: 1–5). Subjects recorded a total of 108 daily records with a median volume of air rebreathed from others of 120.6 [standard error (SE) 8.0] litres per day [Figure 2] with location contributions from own household (48%), school (44%), visited households (4%), transport (0.5%) and other locations (3.5%). While all participants rebreathed air in households every day [59.5 (SE 7.3) litres per day], only 81% (87/108) of recorded days included school attendance, with a mean RAV of 63.1 (SE 5.4) litres per day. Public transport contributed only 0.5% of total RAV of study participants as only 9 adolescents used public transport (12 recorded days) with a mean of 5.8 (SE 0.7) litres in transport per day.

Calculations of mean RAV per hour for each location type were conducted to determine the relative risk in each environment. A mean RAV of 11.5 litres per hour (SE 0.07) was recorded in schools, a mean RAV of 6.3 litres per hour in transport (SE 0.25), a mean RAV of 4.4 litres per hour in households (SE 0.02) and a mean RAV of 5.8 litres in other places (SE 0.09).

**Figure 4:**
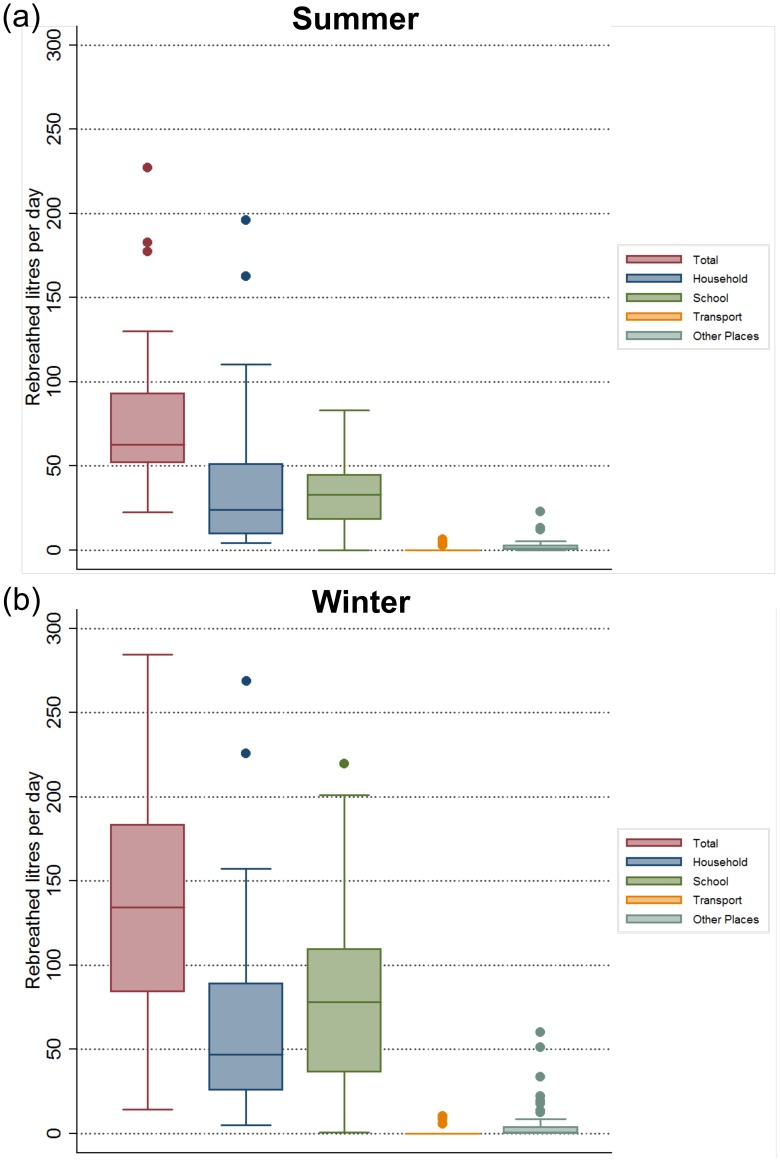
**Figure 4 A**: Volumes of rebreathed air recorded in summer months. The figure shows median, inter-quartile ranges and maximum and minimum volumes of rebreathed air from others for 28 weekdays recorded between November and April together with volume contributions from households, school attendance, transport and various other locations. **Figure 4 B**: Volumes of rebreathed air recorded in winter months. The figure shows median, inter-quartile ranges and maximum and minimum volumes of rebreathed air from others for 63 weekdays recorded between May and October together with volume contributions from households, school attendance, transport and various other locations. N.B. Two outlier values of 395 and 550 litres per day for total volumes and a single outlier of 395 for household volumes are not shown as they exceed the maximal value of the ordinate scale.

Twenty-four adolescents recorded 28 summer weekday records with a mean RAV of 79.2 (SE 9.2) litres per day and 39 adolescents recorded 65 winter weekday records with a mean RAV of 147.1 (SE 10.5) litres per day [Figure 4] (p = 0.008). The mean number of daily contacts in summer (16.9) and winter (14.34) did not differ (p = 0.76). However, the mean time spent indoors was higher in the winter (22.2 hours) than in the summer (19.4 hours) (p<0.001).

In order to establish if alternative locations visited during weekends might contribute to total rebreathed litres, 8 of the subjects completed 15 weekend daily records. Mean weekend litres per day (82.6; SE 20.7) were considerably lower than on weekdays (147.12; SE 10.55), with own (82%) and visited households (10%) the major contributing locations.

In multivariable analysis of the 108 adolescent daily records (Table 1), log RAV per day increased 8% per year of age, increased 14% per added household occupant, increased 17% per additional occupant of sleep space, was 77% higher in winter months and 43% lower in shacks compared with brick dwellings. The median and distribution of RAV at each indoor location are shown in Figure 4, demonstrating increased rebreathing of air in all locations during winter months with greatest impact on household and school. School was the major location of RAV contributing a mean of 77.6 litres per day in winter months.

**Table 1:** Predictors of total log rebreathed litres per day in an unadjusted, multilevel analysis and in a multivariable, multilevel linear regression analysis.

	Unadjusted			Adjusted		
Predictor	ß Coefficient	Exp*	p-value	ß Coefficient	Exp*	p-value
Age *(per year)*	0.05	1.05	0.231	0.08	1.08	0.041
Female *(vs male)*	0.13	1.14	0.468	−0.35	0.70	0.059
Winter *(vs summer)*	0.48	1.62	0.008	0.57	1.77	<0.001
Household *(number)*	0.13	1.14	0.026	0.13	1.14	0.021
Sleep space *(number)*	0.15	1.16	0.041	0.16	1.17	0.022
Shack *(vs brick house)*	−0.44	0.64	0.021	−0.56	0.57	0.002
Weekend *(vs weekday)*	−0.64	0.53	<0.001			

Multivariable model conditional goodness-of-fit: 0.73.

^*^Exponent of ß coefficient, indicating magnitude of change in daily rebreathed volume per unit change in predictor or binary comparator.

## Discussion

The transmission of communicable diseases is understood to be a function of social contact rates and the probability of transmission per contact. Recent studies have illuminated some of the structure and heterogeneity of social contacts [19]–[22], however, there have been few data on the role of the indoor environment for airborne infections which, as Wells and Riley demonstrated, is a key determinant of transmission [12], [13]. Virtually all studies examining environmental risk for tuberculosis focus on households or outbreaks in single environments (e.g. commercial airliners, hospitals). However, studies from Cape Town and Lima have demonstrated that a minority of tuberculosis transmission occurs within households [7], [8]. It has remained unclear where most transmission occurs in endemic settings. In this paper, we demonstrated the measurement of a simple metric—RAV— that integrates social contact and environmental data pertinent to transmission of small particle airborne infections.

We have demonstrated that it is practical to continuously measure ambient CO_2_ concentrations surrounding an individual and thereby estimate the RAV rebreathed from others during normal daily activities. Our approach extends the work of Wells [12], Riley [13], and Rudnick [25] by enabling quantitation of exposure to infected air in multiple non-steady state environments. The sum of the contributions from all visited indoor locations allowed estimation of total daily RAV from others. Adolescents living in a high TB-burdened community recorded very large daily volumes of rebreathed air, such that calculated annual RAV would reach between (IQR) 20 000 to 65 000 litres. Township adolescents had higher RAV compared with our pilot study adults (p<0.0001).

We were able to allocate 93% of rebreathed air to 4 locations: own home, visited homes, transport and work or school. These results corroborate findings of an earlier social mixing study performed in this community in 2010, which reported that 97% of indoor time was spent in these locations. [22] Public transportation use was minimal in this largely local school-attending adolescent population for whom school and household locations contributed the majority of RAV. The daily RAVs were nearly twice as high in the colder winter months than during summer months. The contact rates were comparable between seasons and time spent indoors in winter was only 14% higher, together indicating that increased RAVs were predominantly a result of reduced ventilation, presumably because of need for heat conservation (i.e. closed windows) in cold weather. While there is presently no data on the seasonality of TB infection, our findings may be compatible to the observed seasonality of TB disease in South Africa [31].

While earlier work has examined the role of socio-demographic contact structure in tuberculosis transmission, the role of the indoor environment has not been factored into models of endemic transmission [32].

We propose that the daily RAV may be a useful surrogate marker for the social and environmental components of TB transmission that have been so long recognised but not quantified [9]–[11], [14]. Both the number of individuals within indoor locations and the prevailing environmental ventilation conditions impacts RAV. For an airborne disease such as TB, it is biologically plausible that the total volume exchanged with others would be a major determinant for transmission and acquisition of TB infection [33], which is also consistent with the approaches of Wells [13], Riley [14], Rudnick [25], and others [15]–[20]. The number of secondary active cases generated by an average person with TB in a susceptible population (the basic reproductive number, R_o_) is a fundamental epidemiologic driver of TB epidemics [33]. High-RAV may therefore be a major component maintaining high levels of TB transmission in endemic township populations in Southern Africa [34].

There are several limitations to our study. The major assumption underlying the use of concentrations of inspired CO_2_ as a surrogate for expired air and infection risk is that the dispersion of CO_2_ within an enclosed space reflects the dispersal of infectious particles within that space. According to Stoke's Law, which states that “the nuclei of most droplets atomized indoors shall remain in atmospheric suspension until they are breathed or vented or until they die”, small particles such as TB would not be limited by settling [12]. However, CO_2_ is a highly diffusible gas. CO_2_ decay curves have been widely used for ventilation estimation [35]. Ambient levels of CO_2_ have been long used as a measure of air quality [26], mechanical ventilation control [36] and for airborne disease modelling [25]. CO_2_ concentrations were not sensitive to height of the logger in a room or whether the logger was located on a lanyard or in a waist pocket (data not shown). In order to minimize any direct exposure to exhaled air, the subjects were advised to wear the logger well away from the face and only near the waist. A further caveat to the use of the CO_2_ tracer gas methodology is the assumption that humans are the sole source of CO_2_ in the environments studied. We did require that participants record if there was an obvious alternative source of CO_2,_ such as open flame heat sources. However, other less obvious CO_2_ sources such as the degradation of biological material in earthen floors could possibly impact measurements in informal dwellings [37]. Consistent with prior literature, we assumed that ventilation, rather than CO_2_ absorption or other forms of removal, is the dominant driver of CO_2_ removal from indoor settings [23]. The finding of lower volumes of rebreathed air in shacks compared with brick built structures is compatible with structural leakages which contribute to ventilation, and would also indicate that unsealed, earthen floors did not contribute majorly to household CO_2_ levels. The proportion of RAV from others is also dependent on the accurate recording in the daily diary of person numbers in each indoor location. The precision of recording of small numbers in locations impacts results, but accuracy becomes less important with increased occupancy numbers. If a recording error resulted in 25 persons being recorded as only 20 persons or 50 persons as 40 persons, the errors in rebreathed air would be only 1% and 0.5% respectively. Additionally, the findings of these studies may not be generalizable to other population groups, as the pilot study population was a heterogeneous convenience sample, while the adolescent study was performed in an age-restricted population from a high TB transmission community. Repeated measurements from a small number of schools may underestimate the population variability of RAV. We recorded only up to two daily measurements per person, and additional studies will be required to illuminate the intra-individual variability in rebreathed litres. Finally, the mixed-effect multivariable linear regression analysis was intended to be hypothesis-generating in this study. We had a limited sample size of 93 observations from 63 individuals for the full model. While the design effect was small, there is a possibility of over-fitting, and larger studies are needed to validate these findings.

## Conclusions

In summary, we have demonstrated the practical measurement of CO_2_ over time in a sequence of non-steady state indoor environments, which, combined with data on number of room occupants, enabled the estimation of daily RAV from others. This approach enables comparison of composite social and environmental risk between individuals, settings, and exploration of the determinants of risk (e.g., season). In adolescents residing in a high burden community, this revealed marked variability in RAV between individuals and locations. Future work will be needed to validate this metric by assessing its ability to predict tuberculosis and other respiratory infection risk, which will require larger studies. Continuous monitoring of CO_2_ and subsequent quantification of rebreathed air has great potential as a tool to inform public health interventions targeted at reducing the transmission of airborne respiratory diseases.

## Supporting Information

Data file S1 Rebreathed air volume data.
(CSV)
